# Daily Dietary Selenium Intake in a High Selenium Area of Enshi, China

**DOI:** 10.3390/nu5030700

**Published:** 2013-03-05

**Authors:** Yang Huang, Quanxin Wang, Jin Gao, Zhiqing Lin, Gary S. Bañuelos, Linxi Yuan, Xuebin Yin

**Affiliations:** 1 School of Earth and Space Sciences, University of Science and Technology of China (USTC), Hefei 230026, Anhui, China; E-Mails: rabbit616@mail.ustc.edu.cn (Y.H.); kepu@mail.ustc.edu.cn (Q.W.); gaofei@mail.ustc.edu.cn (J.G.); yuanli@mail.ustc.edu.cn (L.Y.); 2 Advanced Lab for Eco-safety and Human Health, Suzhou Institute of USTC, Suzhou 215123, Jiangsu, China; 3 Environmental Sciences Program, Southern Illinois University, Edwardsville, IL 62026, USA; E-Mail: zhlin@siue.edu; 4 Agricultural Research Service, United States Department of Agriculture, 9611 S. Riverbend Avenue, Parlier, CA 93648, USA; E-Mail: gary.banuelos@ars.usda.gov; 5 Jiangsu Bio-Engineering Research Centre of Selenium, Suzhou 215123, China

**Keywords:** Enshi, selenium, daily dietary intake, hair, selenosis

## Abstract

Enshi is a high selenium (Se) region in Hubei, China, where human selenosis was observed between 1958 and 1963. This study investigated the daily dietary Se intake of residents in Shadi, a town located 72 km northeast of Enshi City, to assess the risk of human selenosis in the high Se area. Foods consumed typically by the local residents and their hair samples were analyzed for total Se concentration. Concentrations of Se in different diet categories were as follows: cereals: 0.96 ± 0.90 mg kg^−1^ DW in rice and 0.43 ± 0.55 mg kg^−1^ DW in corn; tuber: 0.28 ± 0.56 mg kg^−1^ in potato and 0.36 ± 0.12 mg kg^−1^ in sweet potato; vegetables: ranging from 0.23 ± 1.00 mg kg^−1^ in carrot to 1.57 ± 1.06 mg kg^−1^ in kidney bean; animal proteins: 1.99 ± 1.11 mg kg^−1^ in chicken and egg. Based on the food Se concentrations and the daily per-capita consumption, the estimated daily Se intake in Shadi was 550 ± 307 µg per capita. Moreover, the Se concentrations in the hairs of local adult residents were 3.13 ± 1.91 mg kg^−1^ (*n* = 122) and 2.21 ± 1.14 mg kg^−1^ (*n* = 122) for females and males, respectively, suggesting that females might be exposed to higher levels of Se from daily cooking. Although there was no human selenosis occurrence in recent years, the high level of the daily Se intake suggested that the potential risk of selenosis for local residents, especially females, might be a matter of concern.

## 1. Introduction

Selenium (Se) is an essential trace element for animals and humans [1]. It plays an important role in the formation and biofunction of selenoproteins, such as glutathione peroxidase for antioxidant protection of cells [2]. Earlier studies indicated that sufficient Se intake could improve the human immune system and prevent tumor growth by enhancing immune cell activity and suppressing development of blood vessels to the tumor [3,4,5]. Selenium deficiency can lead to human diseases, such as Keshan disease (an endemic cardiomyopathy) and Kashin-Beck disease (a type of osteoarthritis) occurring in Se-deficiency areas in China [6,7]. However, Se can also be toxic to organisms, depending on its chemical species and concentration. Acute Se toxicity is often related to industrial pollution and could cause respiratory, gastrointestinal and/or cardiovascular health problems [8]. Chronic exposure to high levels of Se in food and water could result in hair loss, weak nails, lack of mental alertness, garlic breath odor, excessive tooth decay and discoloration [8]. 

The daily dietary Se intake depends on Se concentrations in foodstuff and the amount of food consumed. Moreover, food Se contents are highly dependent on the soil Se content, as well as the plants’ ability to take up and accumulate Se from the soil [9]. Therefore, human Se intake varies significantly from different consumed food. The estimated Se intake by adults in different countries also varied significantly from 7 to 11 µg day^−1^ in the Keshan disease area [10] to 4990 µg day^−1^ in the selenosis area in Enshi [11]. The recommended dietary allowance (RDA) of Se suggested by the WHO for adults is 55 µg day^−1^ for both males and females [12], while the tolerable upper Se intake level was 400 µg day^−1^ [13]. An earlier study by Yang *et al.* [14] showed that Se homeostasis was disturbed at the Se intake of 750 µg day^−1^ or above, and the symptoms of selenosis occurred at the dietary Se intake of >910 µg day^−1^. In particular, 550 µg Se day^−1^ was considered as the high limit of the Se intake safe rate in high Se areas, such as in Enshi [15]. 

There were 477 cases of human selenosis reported between 1923 and 1988 in different villages of Enshi. For example, there were 283 people suffering from hair and nail loss due to unknown etiology-endemic selenosis in 1963. In the Yutangba village, 19 out of 23 people were affected by high levels of Se, and their livestock died from Se poisoning. As a result, the village was evacuated in the late 1960s. Although no human Se toxicity has been reported in recent years, the symptoms of animal Se toxicity have been observed, including hoof and hair loss [16]. Recently, Qin and collaborators reported that the daily Se intake of the residents from three high-Se areas, Anlejing, Huabei and Yutangba located in Enshi City, was approximately 2144 µg day^−1^, posing a potential chronic selenosis risk for local residents [17]. Selenium concentrations in blood, urine, hair and nail tissues can be used as bio-indicators to evaluate Se accumulation in the human body. Compared with blood or plasma that has been commonly used to determine human Se status, hair can be easily collected and analyzed as a non-invasive bio-indicator [18]. Behne *et al.* [19] investigated 15 male individuals who had taken selenium yeast and/or selenomethionine (SeMet) food supplements in medium doses of 62.5–125 µg Se day^−1^ *versus* high doses of 200–263 µg Se day^−1^ for a time period of one to 24 years, demonstrating that there were highly significant correlations between the Se concentration in muscle and that in whole blood (*r* = 0.90), red blood cells (*r* = 0.91), blood plasma (*r* = 0.87), hair (*r* = 0.89) and toe nails (*r* = 0.85). Indeed, these biological tissues can be good bio-indicators for assessing the Se status in the human body. Moreover, Gao *et al.* [7] reported that the hair Se concentration could be highly related with the level of Se intake and, thus, estimate the risk of Se deficiency in the moderate Se deficiency area of Suzhou, China.

In the present study, to prevent selenosis from happening again in the high Se area, such as Shadi, the commonly consumed foods were collected to determine Se concentrations and to estimate the Se daily intake based on their diet composition, and hair samples were also collected to directly monitor the Se-status in the residents’ bodies. 

## 2. Materials and Methods

### 2.1. Sample Collection

**Figure 1:**
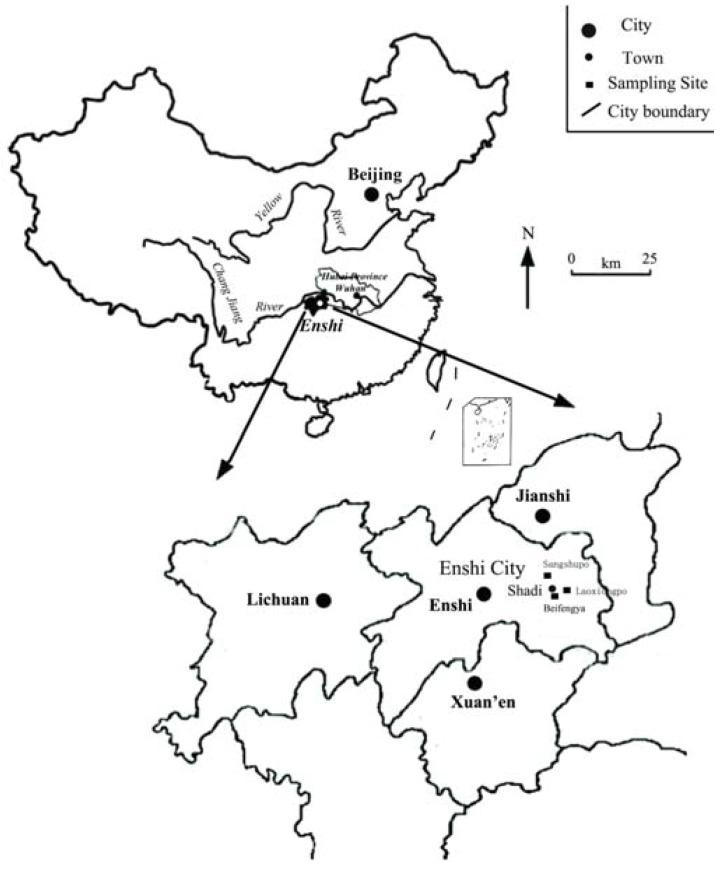
Research location of Shadi town of Enshi city, China.

Samples were collected from Shadi, one of the areas with the greatest number of human selenosis cases (Figure 1). Shadi had high soil Se concentrations with an average of 17.04 mg kg^−1^ dry weight (DW) and a range of 11.26 to 27.50 mg kg^−1^ DW [20]. The sampling sites included three villages, Laoxiongpo, Beifengya and Sangshupo, in the township of Shadi.

Rice (*n* = 66), corn (*n* = 124), tuber (*n* = 18), major types of vegetable (*n* = 69), chicken (*n* = 12) and egg (*n* = 12) samples were purchased from local residents of Laoxiongpo, Beifengya and Sangshupo in Shadi. Meanwhile, 122 hair samples from males and 122 hair samples from females were also collected from those local residents. Approximately 1.0 g of hair samples (about 1–3 cm length) was collected using stainless-steel scissors from the nape (or back of the neck) to the scalp (or occipital) region.

### 2.2. Sample Preparation

The edible parts (*i.e.*, leaves, tuber and bean) of vegetables were washed in distilled water, air-dried and then ground into fine powder to homogenize. For cereal samples, about 200 g of threshed rice were oven-dried at 55 °C, ground using a stainless steel grinder and then stored at 4 °C till chemical analysis. Chicken tissue samples were rinsed in distilled water, air-dried, blended using a pulp refiner and then stored. Hair samples were washedin acetone and distilled water three times, air-dried, cut into small pieces and stored for acid digestion.

### 2.3. Total Se Analysis

Ultra pure water from a Millipore Milli-Q system (Milford, MA, USA) was used for the preparation of all chemical solutions. A 1000 mg L^−1^ Se standard stock solution (Merck, Darmstadt, Germany) was diluted using 5% HCl to 10 mg L^−1^ Se working standard solution. The 1.0% (w/v) NaBH_4_ was prepared using 0.2% (w/v) NaOH solution. All used chemicals (e.g., NaBH_4_, NaOH, concentrated HNO_3_, HCl and HClO_4_) were analytical grade or above and manufactured by Sinopharm Chemical Reagent (Shanghai, China). 

The total Se concentrations in samples were determined according to the method previously reported by Gao *et al.* [7]. In brief, about 0.2 g of samples was digested in HNO_3_–HClO_4_ (4:1, v/v) in a 50 mL conical flask at room temperature overnight. The digestion solution was heated on an electrical hot plate at 100 °C for 1 h, 120 °C for 2 h, 180 °C for 1 h and then kept at 210 °C until a white fume formed. The final volume of the digestion solution was approximately 2 mL. After acid digestion, the solution was cooled to room temperature, then 5 mL of concentrated HCl (12 M) were added to reduce Se (VI) to Se (IV) within at least 3 h. The selenite-Se solution was transferred into a 25 mL volumetric flask and made up to 25 mL by Milli-Q water. The Se concentration was determined by Hydride Generation Atomic Fluorescence Spectrometry (HG-AFS) 9230 (Beijing Titan Instrument Co., Beijing, China), using NaBH_4_ as a reducing agent. Two standard reference materials, shrub leaves (GBW 07603-GSV-2) and hair (GBW 07604-GSV-3), prepared by the National Institute of Minerals of China, were used as the Quality Control/QC samples. The measurement of relative standard deviation (RSD) and the instrument detection limit were 0.76% and 0.08 µg kg^−1^, respectively.

### 2.4. Statistical Analyses

The data were analyzed by the ANOVA procedure at the 0.05 level of significance. The multiple comparison of means was done by the Scott Knott test.

## 3. Results and Discussion

### 3.1. Concentrations of Se in Food

Concentrations of Se in rice and corn harvested from Shadi were 0.96 ± 0.90 mg kg^−1^ DW and 0.43 ± 0.55 mg kg^−1^ DW, respectively (Table 1). Concentrations of Se in corn collected in Shadi were lower than those from the typical selenosis area, Yutangba, with 1.48 ± 1.41 mg kg^−1^ DW [16], Punjab, with a range from 13 to 670 mg kg^−1^ [21], but significantly higher than those from other regions of China, with an average of 0.029 mg kg^−1^ DW [22]. The Se level of rice in Shadi was also significantly higher than those from other areas of China, such as the Keshan disease area, Beijing, Guangzhou, Taiyuan and Suzhou (Table 2).

**Table 1:** Concentrations (mg kg^−1^, dry weight) of selenium in corn and rice samples collected from Shadi, Enshi, China.

	Beifengya	Laoxiongpo	Sangshupo	Average
Rice	0.89 ± 0.46 (*n* = 27)	1.93 ± 1.24 (*n* = 16)	0.36 ± 0.19 (*n* = 23)	0.96 ± 0.90 (*n* = 66)
Corn	0.33 ± 0.31 (*n* = 33)	0.58 ± 0.67 (*n* = 67)	0.13 ± 0.15 (*n* = 24)	0.43 ± 0.55 (*n* = 124)

**Table 2:** Concentrations of selenium in different foods produced in different regions of China (mg kg^−1^, wet weight).

	Rice ^a^	Soybean	Tuber	Leaf vegetables	Chicken	Egg
Keshan disease area [23]	0.006	0.012	- ^b^	0.00007	0.034	0.484
Beijing [24]	0.049	0.070	0.003	0.020	-	0.213
Guangzhou [25]	0.058	0.085	0.005	0.006	0.108	0.237
Taiyuan [26]	0.037	0.073	0.008	0.011	0.174	0.152
Suzhou [7]	0.024	-	0.004	0.003	0.112	0.152
Shadi (This study)	0.96	0.71	0.33	0.72	1.72	2.26

^a^ Se concentration expressed on a dry weight basis. ^b^ not detected.

Concentrations of Se in different diet categories (Table 3) were as follows: (1) Tuber: 0.28 ± 0.56 mg kg^−1^ in potato and 0.36 ± 0.12 mg kg^−1^ in sweet potato; (2) Vegetables: ranging from 0.23 ± 1.00 mg kg^−1^ in carrot to 1.57 ± 1.06 mg kg^−1^ in kidney bean; (3) Animal proteins: 1.99 ± 1.11 mg kg^−1^ in chicken and egg. Due to significant variation in soil Se distribution, concentrations of Se in cereals also varied significantly, showing that cereals from Laoxiongpo had the highest levels of Se accumulation. Therefore, compared with other regions of China (Table 2), foods produced in Shadi have higher Se concentrations. 

**Table 3:** Concentrations of selenium in foods grown in Shadi, Enshi, China.

Food	Se Concentration (mg kg^−1^, wet weight)			
	N	Mean	SD^ a^	Range
*Vegetables*				
Carrot	10	0.23	1.00	0.07–1.60
Garlic	8	0.53	0.22	0.33–1.08
Hyacinth bean	8	0.57	1.22	0.11–3.75
Chinese cabbage	10	0.72	0.92	0.31–2.73
Pumpkin	9	0.76	1.21	0.31–3.22
Eggplant	9	1.04	1.06	0.43–3.21
Kidney bean	5	1.57	1.06	0.41–4.14
Soybean	10	0.71	0.54	0.46–1.37
*Tuber*				
Potato	7	0.28	0.56	0.04–1.07
Sweet potato	11	0.36	0.12	0.06–0.89
*Animal source foods*				
Chicken	12	1.72	1.04	1.07–2.94
Egg	12	2.26	1.12	0.94–4.21

^a^ SD = Standard Deviation.

### 3.2. Daily Dietary Se Intake

Previous investigation on the daily diet of residents in Shadi showed that it comprised 315 g of cereals, 230 g of tubers, 135 g of vegetables and 45 g of animal proteins [27]. Based on the composition of residents’ daily diet and the Se concentrations in the selected food materials in Section 3.1, the estimated daily Se intake per capita was 527 ± 212 µg in Beifengya, 833 ± 392 µg in Laoxiongpo and 370 ± 175 µg in Sangshupo (Table 4). The calculated average daily Se intake in the Shadi area was 550 ± 307 µg per capita, which was equal to the suggested toxic Se intake dose of 550 µg for adults in a high Se area [15], but lower than the daily dietary Se intake of 910 µg at which the selenosis symptoms had been observed. Therefore, even in Laoxiongpo, no visible Se poisoning symptoms were observed. Moreover, It was possible that after long-term exposure to a high Se through diet, local residents might be able to tolerate higher Se concentrations [28,29]. The change in the consumption pattern might be another important reason to explain the phenomenon of no human selenosis occurrence. In general, organic Se forms were safer than inorganic Se [30]. The rice Se, the major source of selenium intake for local residents, was dominated by SeMet with less methylselenocysteine (SeMeSeCys) and inorganic Se [31]. However, the daily Se intake of residents in the present study were still remarkably higher than those from most other countries/areas, e.g., Europe with 30–100 µg day^−1^, North America with 60–220 µg day^−1^ [32], Japan with 140–178 µg day^−1^ [33] and comparable with that from the high-Se areas of Punjab, India, with 475–632 µg d^−1^, where clinical symptoms of selenium toxicity were observed in some of the local residents [34]. Therefore, the potential risk of selenosis for local residents may be a matter of concern. Qin *et al.* [17] reported that the daily Se intake of the residents from three other high-Se areas, Anlejing, Huabei and Yutangba, located in Enshi City, was approximately 2144 µg day^−1^, which was greatly higher than that from Shadi in this study. The possible reason was that the foods were collected from different sites, and the distribution of Se in soils was significantly uneven. In the High-Se-Toxicity villages, soil Se concentrations ranged from 2.736 to 27.5 mg kg^−^^1^, with the mean of 9.46 mg kg^−^^1^ [16].

**Table 4:** Daily Se intake and hair Se content of local residents from the three villages.

	Daily Se Intake (mg kg^−1^)	Hair Se (mg kg^−1^)
Beifengya	527 ± 212	2.40 ± 1.43
Laoxiongpo	833 ± 392	3.07 ± 1.52
Sangshupo	370 ± 175	2.48 ± 2.00

### 3.3. Concentrations of Se in Hair

The hair Se concentrations in local residents were 2.21 ± 1.14 mg kg^−1^ (*n* = 122) for males and 3.13 ± 1.91 mg kg^−1^ (*n* = 122) for females. The average Se concentration in female hair was significantly higher than that in male hair (*p* < 0.05), and the high Se concentrations were frequently observed in adult female hair samples, which might be a result from the fact that women had higher levels of exposure to Se from their daily cooking. Actually, local high Se coals were often used for in-door cooking in the present study area. Earlier studies showed that hair Se content from people with chronic selenosis was 32.2 mg kg^−1^, but 3.7 mg kg^−1^ from people without selenosis in the same high Se area [11]. In this study, the majority of the hair samples contained Se concentrations of <8.2 mg kg^−1^, except for some female hair samples. 

Concentrations of Se in hair samples collected from the three villages was 2.40 ± 1.43 mg kg^−1^ in Beifengya, 3.07 ± 1.52 mg kg^−1^ in Laoxiongpo and 2.48 ± 2.00 mg kg^−1^ in Sangshupo (Table 4). Although the daily Se intake of Beifengya (527 ± 212 µg) was higher than that of Sangshupo (370 ± 175 µg), the hair Se concentration in Beifengya was significantly lower than in Sangshupo (*p* < 0.05). Meanwhile, there was no significant (ANOVA) correlation between the hair Se and the rice Se (the major source of selenium intake for local residents). It was possible that the level of Se in hair was dependent on the environmental conditions and the Se intake during a long time period. Although, in the introduction, it was noted that in one study, Se in muscle was correlated even more highly with hair Se than with plasma Se. These findings suggested that Se concentration in hair might not be a good bio-indicator for daily Se intake. 

### 3.4. Changes of Se Content in Local Cereal in the Past 40 Years

To figure out the change trends, data on Se contents in local corn (Table 5) from 1963 to 2012 were compiled [35]. In 1963, when selenosis happened, the Se concentration in corn in Enshi had the highest Se concentration of 33.47 mg kg^−1^ DW [36]. The average Se concentration in corn dramatically reduced to 8.66 mg kg^−1^ in 1966, three years after the reported selenosis [37]. In the 1980s, the Se concentrations in corn decreased to 4.17–14.07 mg kg^−1^ DW [36,38] and remained relatively stable throughout the early 1990s, with a Se concentration range of 5.95–6.47 mg kg^−1^ in corn [39,40]. However, in the later 1990s, the Se concentrations decreased to 1.38–1.48 mg kg^−1^ during the late 1990s [16,35]. In the present study, the average Se concentration in corn was only 0.43 mg kg^−1^. Similar trends were found in other plant species, such as rice, bean, carrot, garlic, hyacinth bean, Chinese cabbage, pumpkin, eggplant, kidney bean and potato. Such a substantial decrease in Se accumulation in crops in Enshi since 1963 was a result of reducing the utilization of Se-coal stone ash (up to 8390 mg kg^−1^) [41] on agricultural lands. Meanwhile, owing to the loss of Se volatilization, selenium in soil had undergone a natural process of depletion over the past 40 years [42]. As shown in Table 5, Se concentrations in soil remain almost the same as before, indicating that soluble Se had been released and the bioavailability of Se in soil had decreased.

In 1966, the daily Se intake in Enshi was 4990 µg with a range of 320 to 6690 µg [11]. Yin *et al.* (1996) indicated that the value reduced to 1338 µg in 1985 [40]. The present study showed that the daily Se intake significantly reduced to 550 µg, but the average Se daily intake value still reached the recommended maximum safe intake. Consequently, more attention should be paid on preventing human selenosis (or Se poisoning) in the present study area. Furthermore, Se content in blood and plasma need to be determined to accurately diagnose the Se-status of local residents in future studies.

**Table 5:** The variation of Se in soil and corn in the past 40 years.

Se in Soil (mg/kg dry weight)		Se Content (mg/kg dry weight)		Sampling Time (year)	References
N	mean (min-max)	N	mean (min-max)		
	6.83		33.47	1963	[36]
6	7.68(0.08–45.5)	5	8.66 (0.5–44.0)	1966	[37]
4	3.45 (1.92–4.98)	4	14.07	1987	[36]
9	5.48 (0–11.89)		4.17 (0.77–7.57)	1989	[38]
28	4.06 (2.82–5.30)	130	6.47 (2.18–10.76)	1992	[39]
			5.95 (4.40–7.50)	1995	[40]
5	4.99 (2.61–7.37)	5	1.38 (0.182–5.60)	1996	[16]
150	4.75 (0–12.18)	20	1.48 (0.07–2.89)	1999	[35]
		124	0.37 (0–0.79)	2010	This study

## 4. Conclusions

Based on diet composition, the Se daily dietary intake of residents of Shadi was calculated as 550 ± 307 µg per capita, equal to the suggested toxic Se intake dose. Moreover, the Se concentrations in female hair (3.13 ± 1.91 mg kg^−1^) were significantly higher than that in male hair (2.21 ± 1.14 mg kg^−1^), suggesting that the high levels of Se exposure for women would be an important matter of concern in future Se risk assessment in Shadi. Although concentrations of Se in foods and Se daily dietary intake have greatly decreased over the past 40 years, the potential threat of selenosis to local residents still exists. More research is needed to further assess the Se-status of local residents, such as Se in plasma.
